# Supplementary material for: Machine Learning-Based Pattern Recognition of Risk Factors for Low Back Pain among Adolescent Cricket Players in Dhaka City
Source: PLOS Digit Health. 2026 Apr 17;5(4):e0001369. doi: 10.1371/journal.pdig.0001369 (PMC13089699; doi:10.1371/journal.pdig.0001369)
Supplement: Attachment — Submitted filename: Rebuttal Letter.docx [file pdig.0001369.s006.docx]

To,

The Editor, Reviewers,

PLOS Digital Health

Date: 14^th^ October, 2025

**Subject: Addressing Editor/Reviewer Comments for PDIG-D-25-00567**

Dear Editor, Reviewers,

Many thanks to you for your helpful feedback. We are sorry that it took so long for us to revise. We have now tried to address all your concerns to the best of our abilities. Kindly help us with your further concerns (if any) to improvise our paper. Below we are providing our responses to the comments and actions that we took to address them. The responses are highlighted in blue.

Thank you.

Kind regards,

Authors of PDIG-D-25-00567

*Peer Review Analyze: Machine Learning-Based Pattern Recognition of Risk Factors for Low Back Pain among Adolescent Cricket Players in Dhaka City*

*Joynal Abedin Imran, Pradip Kumar Saha, Marzana Afrooj Ria, Syeda Saika Sarwar, Jannatul Ferdous Konok*

PDIG-D-25-00567

Machine Learning-Based Pattern Recognition of Risk Factors for Low Back Pain among Adolescent Cricket Players in Dhaka City

PLOS Digital Health

Dear Dr. Marzana Afrooj Ria,

Thank you for submitting your manuscript to PLOS Digital Health. After careful consideration, we feel that it has merit but does not fully meet PLOS Digital Health's publication criteria as it currently stands. Therefore, we invite you to submit a revised version of the manuscript that addresses the points raised during the review process.

Please submit your revised manuscript within 60 days Nov 15 2025 11:59PM. If you will need more time than this to complete your revisions, please reply to this message or contact the journal office at [digitalhealth@plos.org](mailto:digitalhealth@plos.org). When you're ready to submit your revision, log on to <https://www.editorialmanager.com/pdig/> and select the 'Submissions Needing Revision' folder to locate your manuscript file.

Please include the following items when submitting your revised manuscript:

- A rebuttal letter that responds to each point raised by the editor and reviewer(s). You should upload this letter as a separate file labeled 'Response to Reviewers'. This file does not need to include responses to any formatting updates and technical items listed in the 'Journal Requirements' section below.
  - Included
- A marked-up copy of your manuscript that highlights changes made to the original version. You should upload this as a separate file labeled 'Revised Manuscript with Track Changes'.
  - Included
- An unmarked version of your revised paper without tracked changes. You should upload this as a separate file labeled 'Manuscript'.
  - Included

If you would like to make changes to your financial disclosure, competing interests statement, or data availability statement, please make these updates within the submission form at the time of resubmission. Guidelines for resubmitting your figure files are available below the reviewer comments at the end of this letter.

We look forward to receiving your revised manuscript.

Kind regards,

Cleva Villanueva, M.D., Ph.D.

Academic Editor

PLOS Digital Health

Cleva Villanueva

Academic Editor

PLOS Digital Health

Leo Anthony Celi

Editor-in-Chief

PLOS Digital Health

[orcid.org/0000-0001-6712-6626](http://orcid.org/0000-0001-6712-6626)

Journal Requirements:

1. Please provide an Author Summary. This should appear in your manuscript between the Abstract (if applicable) and the Introduction, and should be 150–200 words long. The aim should be to make your findings accessible to a wide audience that includes both scientists and non-scientists. Sample summaries can be found on our website under Submission Guidelines:

<https://journals.plos.org/digitalhealth/s/submission-guidelines#loc-parts-of-a-submission>

We have now included Author Summary.

2. We note that there is identifying data in the Supporting Information file < Data.csv>. Due to the inclusion of these potentially identifying data, we have removed this file from your file inventory. Prior to sharing human research participant data, authors should consult with an ethics committee to ensure data are shared in accordance with participant consent and all applicable local laws.

Data sharing should never compromise participant privacy. It is therefore not appropriate to publicly share personally identifiable data on human research participants. The following are examples of data that should not be shared:

 -Name, initials, physical address

-Ages more specific than whole numbers

-Internet protocol (IP) address

-Specific dates (birth dates, death dates, examination dates, etc.)

-Contact information such as phone number or email address

-Location data

-ID numbers that seem specific (long numbers, include initials, titled “Hospital ID”) rather than random (small numbers in numerical order)

Data that are not directly identifying may also be inappropriate to share, as in combination they can become identifying. For example, data collected from a small group of participants, vulnerable populations, or private groups should not be shared if they involve indirect identifiers (such as sex, ethnicity, location, etc.) that may risk the identification of study participants.
 
Additional guidance on preparing raw data for publication can be found in our Data Policy (<https://journals.plos.org/plosone/s/data-availability#loc-human-research-participant-data-and-other-sensitive-data>) and in the following article: <http://www.bmj.com/content/340/bmj.c181.long>.
 
Please remove or anonymize all personal information (<specific identifying information in file to be removed>), ensure that the data shared are in accordance with participant consent, and re-upload a fully anonymized data set. Please note that spreadsheet columns with personal information must be removed and not hidden as all hidden columns will appear in the published file.

We thank the editor for highlighting this important issue. As the study involves a vulnerable population (adolescents aged 11-19 participating in cricket in Dhaka City, Bangladesh), we prioritized participant privacy in line with PLOS Digital Health's data policy, the referenced BMJ article on data preparation, and ethical guidelines. We consulted our institution's ethics committee (Dhaka Medical College Ethics Review Board), which confirmed that the revised dataset aligns with participant consent (which allowed de-identified data sharing for research purposes) and applicable local laws (e.g., Bangladesh's Digital Security Act and health data protection norms). No personally identifiable information was collected in the original survey beyond what is necessary for analysis, and consent forms emphasized data anonymization for publication.

To address the concerns:

We reviewed the dataset for direct identifiers (e.g., names, addresses, dates, contacts, IPs, specific IDs) – none were present.

For indirect identifiers, we identified potential risks from precise continuous variables (e.g., exact age, height, weight, BMI, family income, sports experience years, practice/warm-up/cool-down durations), which could combine with categorical data (e.g., sex, education level, playing position) to re-identify individuals in this niche group.

Actions taken: We removed all raw precise columns (Q1_Age, Q4_Family_income, Q5_Height, Q6_Weight, Q7_BMI, Q9_Sports_Exp, Q10_Practice_hour, Q12_W_dur, Q14_Cool_Dur). These were replaced by retaining only their pre-existing categorical/binned versions (e.g., Age_cat, Mon_Income_Cat, BMI_Cat), which are broader and less identifying while preserving the data's utility for replicating findings.

The Id_No column (sequential 1-450) was retained as it is non-specific and serves as a random participant index, consistent with the policy's allowance for "small numbers in numerical order."

No columns were hidden; they were fully removed.

The dataset now has 16 columns (down from 25), with all entries anonymized. Empty/missing values (e.g., spaces in some fields) were preserved as is, but since affected columns were dropped, they no longer pose issues.

This anonymization ensures no compromise to privacy, as combinations (e.g., age category + sex + BMI category) are unlikely to uniquely identify individuals in a sample of 450 from a large urban population.

If the reviewer comments include a recommendation to cite specific previously published works, please review and evaluate these publications to determine whether they are relevant and should be cited. There is no requirement to cite these works unless the editor has indicated otherwise.

**Additional Editor Comments (if provided):**

Reviewer #1: Abstract: Needs clearer statement of feature correlates and explicit mention of study limitations. Absence of the severe pain category reduces the model’s capacity to triage urgent cases.

Introduction: Should include local statistics on LBP in Bangladesh cricketers, acknowledge genetic predisposition as a factor, and cite comparative studies (traditional vs. ML approaches). Needs references to existing ML work on LBP and discussion of ML’s achievements in other contexts.

Methods: Adequate sample size (n=450) and sound justification for regression models. However, purposive sampling may cause bias, reliance on self-reported data is limiting, no feature selection analysis mentioned, unclear categorical variable encoding, and absence of external validation.

Results: Well presented, but tables could be shorter. Authors should discuss the clinical implications of low recall in Class 2 (66%).

Discussion: Strong comparative analysis, but requires deeper discussion on clinical significance of Class 2 low recall and external validation for generalizability.

Overall: A relevant study with strong potential, but would be significantly strengthened by external validation, refined methodology, and clearer justification in introduction.

Reviewer #2: Introduction: Too long and should be condensed to fewer than 400 words without omitting important content.

Methods: Missing inclusion/exclusion criteria (e.g., training duration per week required for eligibility). Avoid reporting model performance in the Methods section—this belongs in Results.

Results: First paragraph should instead describe participant demographics. Authors should follow a standardized checklist (e.g., STROBE) for reporting cross-sectional studies.

Formatting: Inconsistent numerical reporting (percentages in text, decimals in tables) should be standardized.

Overall: Main issues relate to manuscript organization, clarity, and adherence to reporting standards.

Reviewer #3: Overall Premise: Interesting use of ML for a low-cost solution, but key conceptual issues remain.

Main Concerns:

Lack of clarity on how athletes were classified into “no, mild, and moderate LBP” despite existing literature.

No discussion on potential collinearity of variables, which may compromise model stability.

Risk factors identified are already well-established in prior studies—unclear what the novel contribution is.

Practical application of the algorithm in real-world settings is not addressed.

Introduction: Some references inappropriate (e.g., citation of qualitative/lived experience study as demographic evidence). Authors should use more robust sources (large cohort studies, meta-analyses, RCTs). Questionable logical connections in cited arguments.

Methods: Lack of clarity on who categorized LBP severity, which standard was used, and what reference supports it.

Overall: Raises doubts about novelty, validity of references, and real-life applicability of findings.

[Note: HTML markup is below. Please do not edit.]

**Reviewers' comments:**

Reviewer's Responses to Questions

**Comments to the Author**

1. Does this manuscript meet PLOS Digital Health’s [publication criteria](https://track.editorialmanager.com/CL0/https:%2F%2Fjournals.plos.org%2Fdigitalhealth%2Fs%2Fjournal-information%23loc-criteria-for-publication/1/010f019950dc805e-d087e8c3-7644-4e1e-9026-1184406b8532-000000/uzHQb_86e31DMKkU1qRQdvsHMlS612QK4PZ5rQlByGo=228)? Is the manuscript technically sound, and do the data support the conclusions? The manuscript must describe methodologically and ethically rigorous research with conclusions that are appropriately drawn based on the data presented.

Reviewer #1: Partly

Reviewer #2: No

Reviewer #3: Yes

2. Has the statistical analysis been performed appropriately and rigorously?

Reviewer #1: No

Reviewer #2: Yes

Reviewer #3: No

3. Have the authors made all data underlying the findings in their manuscript fully available (please refer to the Data Availability Statement at the start of the manuscript PDF file)?

The [PLOS Data policy](https://track.editorialmanager.com/CL0/https:%2F%2Fjournals.plos.org%2Fdigitalhealth%2Fs%2Fdata-availability/1/010f019950dc805e-d087e8c3-7644-4e1e-9026-1184406b8532-000000/JLBA5pAIoJdAPAvloLtmbq328cn5bOvFVVTrBgKN9zQ=228) requires authors to make all data underlying the findings described in their manuscript fully available without restriction, with rare exception. The data should be provided as part of the manuscript or its supporting information, or deposited to a public repository. For example, in addition to summary statistics, the data points behind means, medians and variance measures should be available. If there are restrictions on publicly sharing data—e.g. participant privacy or use of data from a third party—those must be specified.

Reviewer #1: Yes

Reviewer #2: No

Reviewer #3: Yes

4. Is the manuscript presented in an intelligible fashion and written in standard English?

PLOS Digital Health does not copyedit accepted manuscripts, so the language in submitted articles must be clear, correct, and unambiguous. Any typographical or grammatical errors should be corrected at revision, so please note any specific errors here.

Reviewer #1: No

Reviewer #2: Yes

Reviewer #3: Yes

5. Review Comments to the Author

Please use the space provided to explain your answers to the questions above. You may also include additional comments for the author, including concerns about dual publication, research ethics, or publication ethics. (Please upload your review as an attachment if it exceeds 20,000 characters)

Reviewer #1: Review of Machine Learning-Based Pattern Recognition of Risk Factors for Low Back Pain among Adolescent Cricket Players in Dhaka City

Abstract

A Relevant study with clear focus

The abstract section will be more helpful if the feature correlates were clearly stated.

The study acknowledged the absence of the fourth class (Severe pain category) impacting the depth and rigor of the study.

(The model developed by this study may have reduced capacity to triage LBP and determine cricket players who need urgent intervention especially the severe pain category). The circumstances is understandable albeit.

The limitations of this study should be summarized in the abstract.

We have now revised the content as per suggestion.

Actions Taken: Revised Abstract to include explicit correlates (e.g., female gender AOR=2.52, inconsistent warm-up AOR=12.48-14.07), noted severe pain exclusion's effects on triage/rigor, and added a concise prime limitation sentence covering exclusion of severe pain category due to insufficient data which reduces the model’s capacity to triage urgent cases requiring immediate intervention.

Introduction

The authors should add compelling local statistics demonstrating the severity of LBP amongst cricketers in Bangladesh or is there a lack of studies exploring this ? Which then may be a solid justification for the existence of this study.

The study acknowledged the strong possibility of the under-reportage of LBP in Bangladesh

There is lack of specific LBP statistics for adolescent in Dhaka. We have now added justification as per suggestion.

Action taken: Added local statistics reporting high musculoskeletal pain prevalence (up to 78.3%) among adolescents in Dhaka, including lower back commonly affected (Alam et al., 2024; Rana et al., 2025).

The authors did not acknowledge the fact that genetic predisposition also account for LBP, it isn’t all mechanical.

We agree and have added this aspect.

Actions Taken: Included genetic predispositions (heritability 30-50%) with citations (Battié et al., 2007; Livshits et al., 2011) in the Introduction to highlight multifactorial etiology.

The study will benefit from citation of comparative studies where traditional methods have shown limited capacity which will strengthen the justification of Machine learning for this study.

We have incorporated this.

Actions Taken: Cited limitations of traditional methods (e.g., poor predictive validity, nonlinearity issues from Trompeter et al.; Karran et al., 2017) in the Introduction to justify ML.

”There are no known predictive models with ML to be used in screening LBP in adolescent cricketers in Dhaka “ Other studies on LBP among cricketers using ML for predictive analysis should be cited to justify the need to carry out this study in Bangladesh

Also, the study should highlight statistically how the performance and effectiveness of ML in predicting the occurrence of LBP amongst cricketers in other places, what achievements so far? Has it reduced the occurrence?

Thank you for this insightful suggestion, which strengthens the justification for our study's novelty. Upon conducting a thorough literature search (including PubMed, Scopus, and web-based queries for "machine learning predictive models for low back pain in cricketers"), we confirmed that no prior studies have developed ML-based predictive models specifically for LBP screening in cricketers, particularly adolescents in Dhaka or broader Bangladesh contexts.

Actions Taken: Confirmed no cricket-specific ML models via search; Already added citations to general ML studies (e.g., accuracies 83-92%) for comparison in Discussion; emphasized gap for Bangladesh/adolescent cricketers.

Materials and Method:

n = 450 seems adequate for this study

Ethical consideration clearly stated

Purposive sampling may introduce selection bias

The study demonstrated heavy reliance on self reported data and also, no mention of feature selection analysis

Regarding the reliance on self-reported data, we acknowledge this as a common approach in epidemiological and sports injury research for capturing subjective experiences like pain severity (e.g., via Numeric Pain Rating Scale NPRS), but it may introduce recall or social desirability bias.

Action taken: to address this, we have added a statement in the Methods section noting this potential limitation and in the Limitations section for broader discussion. On feature selection, no formal automated methods (e.g., recursive feature elimination) were employed, as variables were pre-selected based on domain expertise and prior literature to ensure clinical relevance and avoid overfitting in our moderate-sized dataset (n=450); this rationale has been explicitly added to the Methods summary.

How were the categorical variables encoded?

Categorical variables were encoded using a hybrid approach in which ordinal and binary features such as Age category, Sex, and Education level were transformed using label encoding to preserve their inherent order, while nominal features were transformed using one-hot encoding to maintain interpretability and ensure compatibility with machine learning algorithms including SVM, as implemented in scikit-learn. This approach offers technical advantages by reducing feature dimensionality, preserving meaningful ordinal relationships, preventing the introduction of spurious ordinal assumptions for nominal variables, and improving both computational efficiency and predictive performance across diverse algorithms.

The study could have benefited from an external validation

While external validation would indeed strengthen generalizability, it was not feasible due to resource constraints in this initial study; we have noted this as a key limitation and recommended it as a priority for future work in the Discussion and Limitations sections. These revisions improve methodological clarity without altering the core findings.

The method section provided a good justification for the use of the regression models and dropping the deep learning model considering the small sample size.

The inclusion of the regression models for comparative analysis makes this study more robust

The use of a balanced dataset, cross validation and grid search reduces overfitting which makes the study more robust.

Results.

Clear presentation of performance metrics of the models employed for the study

Table can be more abridged

For class 2, a low recall value of 66% and F1 value of 77% was achieved by the SVM classifier.

Can the authors address the clinical implication of this?

Action taken: Regarding the SVM's low recall (66%) and F1-score (77%) for Class 2 (moderate pain), we have addressed the clinical implications by adding a dedicated sentence in the Results section, noting that this indicates potential under-detection of moderate cases, which could delay interventions like physiotherapy or training modifications, thereby increasing risks for progression to severe LBP or chronicity in adolescents. We further expanded on this in the Limitation section.

The study has a visual appeal (Visualization of results)

Discussion

This chapter features a robust analysis of the performance metrics and good comparative analysis of the study with other studies on LBP.

The authors should have a more robust discussion on the clinical significance of the class 2 low recall value and what steps to take to mitigate it.

Action taken: To enhance the robustness of the discussion on the clinical significance of the SVM's low recall (66%) and F1-score (77%) for Class 2 (moderate pain), we have expanded the relevant paragraph to explicitly address implications, such as the risk of under-detection leading to delayed interventions (e.g., physiotherapy or load management), which could exacerbate progression to severe or chronic LBP in adolescents, potentially affecting long-term athletic participation and quality of life.

There is a need for the authors to conduct an external validation of their model to assess generalizability and performance on unseen data.

Regarding external validation, while not conducted in this study due to resource limitations, we have explicitly acknowledged this as a key limitation and recommended it as a priority for future research to evaluate generalizability and performance on unseen data from diverse cohorts (e.g., rural Bangladeshi or international cricketers).

Overall: A very germane study that would be more impactful with an external validation and more refined methodology.

Reviewer #2: - Introduction is too long and can be summarized without deletion of important points. please re-write the introduction in less than 400 words.

We have now revised the content as per suggestion.

Actions Taken: Condensed Introduction to 289 words, retaining key points on prevalence, risks, gaps, and ML justification.

- please report the inclusion and exclusion criteria; for example, what was the minimum training duration per week needed to enter the study.

To address the inclusion and exclusion criteria, we have revised the Methods section to explicitly state them: inclusion required adolescents aged 11-19 years actively participating in cricket training or matches for at least 5 hours per week (to ensure sufficient exposure as active players), with no recent (past 6 months) major injuries unrelated to LBP; exclusion encompassed those with congenital spinal deformities, recent surgical interventions, or unwillingness to participate.

- please avoid reporting the performance of different models in method section. for example:

"Although it offered interpretable baseline results, it was less effective than more robust classifiers like SVM in capturing complex relationships between low back pain and its contributing factors". this information belongs to result section

Regarding performance reporting in the Methods, we have removed all comparative phrases, such as the example provided ("Although it offered interpretable baseline results, it was less effective than more robust classifiers like SVM..."), relocating any relevant interpretations to the Results section to maintain methodological focus on descriptions and justifications only.

- The first paragraph of results belongs to reporting the demographics of included subjects. please use a standard check list such as "STROBE Statement—Checklist of items that should be included in reports of cross-sectional studies"

For the Results section, we have restructured the first paragraph to explicitly report participant demographics and flow, aligning with the STROBE checklist for cross-sectional studies.

Actions Taken: Restructured Results opening to align with STROBE

- It is advised to report the numbers in one format. in this manuscript the numbers are reported as percentages in text but as decimals in tables.

To ensure consistent formatting, we have standardized all numerical reports to percentages (e.g., accuracy 0.876 → 87.6%) in both text and tables, enhancing readability and uniformity throughout the manuscript.

We thank Hamidreza Ashayeri for these practical suggestions, which have improved the manuscript's clarity, structure, and adherence to reporting standards.

Reviewer #3: The authors provided an interesting premise on using machine learning to provide low cost solution for assessing low back pain (LBP) risk among young cricket athletes in Dhaka. Specific cricket fast bowling techniques are well known to increase risk of neural arch fracture due to the biomechancial load on the spine. My main concern is that the authors did not discuss how they even classify these athletes into no, mild and moderate LBP, when multiple reviews have been done regarding this subject. Another main issue is lack of discussion how some of the variables used in this study might be highly correlated, which is known to cause instability on a predictive model. Last but not least, the risk factors identified in this study had previously been discovered in prior study, so what is the added value of this study? How would the algorithm be used in real life setting?

We thank Reviewer #3 for the positive assessment of our study's premise as an interesting, low-cost ML-based solution for LBP risk assessment in young Dhaka cricketers, and for raising these important concerns, which have prompted valuable revisions to enhance methodological clarity and interpretive depth. Regarding the classification of athletes into no, mild, and moderate LBP categories, we have revised the Methods section to explicitly detail our approach: severity was self-reported via the Numeric Pain Rating Scale (NPRS), a validated tool where participants rate pain from 0 (no pain) to 10 (worst imaginable), with categories defined as no pain (0), mild (1-3), and moderate (4-6), excluding severe (7-10) due to insufficient cases; this was performed by the research team based on established standards. On potential variable correlations causing model instability, we have added variance inflation factors (all VIF <5) , confirming no significant multicollinearity per standard thresholds; this is further discussed in the Limitations for cautious interpretation of interrelated factors. Lastly, while some risk factors (e.g., prior LBP, inconsistent warm-ups) have been identified in prior studies, our study's added value lies in being the first to integrate them into an ML predictive model (SVM achieving 87.6% accuracy) tailored to adolescent cricketers in Dhaka, a low-resource context lacking such tools; this enables data-driven, context-specific prevention amid unique local factors like urban club training. In real-life settings, the algorithm could be deployed via mobile apps or clinic software for rapid risk screening by coaches or physiotherapists, facilitating personalized interventions (e.g., warm-up protocols) to reduce LBP incidence, future validation will refine this translational potential. These revisions bolster the manuscript's rigor without altering core findings.

Actions Taken: Detailed Numeric Pain Rating Scale (NPRS) classification in Methods with review citations; added VIF analysis in Results/Limitations; emphasized added value (first cricket & in Dhaka ML model) and real-life applications in Discussion.

Below are more detailed comments on this paper:
Intro

- Line 5: up to 30% of adolescents affected. The cited reference 2 is not a demographic study but qualitative study regarding young athlete’s lived in experience. not appropriate for this statement

We have now revised the content as per suggestion.

Actions Taken: Replaced ref [2] with systematic review (Wall et al., 2022) for prevalence in Introduction.

- Ref 4 narative review, ref 7 is case report, ref 8 is scoping review. Can you provide more objective sources of information such as large demographic studies, network meta analysis or randomized controlled trials?

We have now revised the content as per suggestion.

Actions Taken: Supplemented refs with Systematic Review, meta-analyses (Trompeter et al.; Karran et al., 2017) and cross-sectional studies (Alam et al., 2024; Rashaduzzaman et al., 2020) in Introduction.

- Line 34: How does argument 6 supports the argument about the scarcity of biomechanical assessment in Dhaka?

Upon review, we recognize this citation was misplaced, as it addresses general cricket biomechanics rather than Dhaka-specific gaps; to clarify, we have rephrased the sentence to emphasize the overall lack of localized studies in Bangladesh, citing Sany et al., 2021 as cross-sectional demographic studies on LBP risks in Bangladeshi populations, which confirm the scarcity of cricket-focused biomechanical assessments in this context, justifying our study's novelty.

Methods

- Line 60-64: Who categorized the LBP severity, based on what standard and what is the related reference used to use this standard?

To address this, we have revised the section to specify that severity was categorized by the research team based on self-reported participant ratings using the Numeric Pain Rating Scale (NPRS), a standard 0-10 tool where no pain=0, mild=1-3, moderate=4-6 (excluding severe=7-10 due to insufficient cases), supported by references including Karcioglu et al. (2018) a systematic review for its validity.

- Line 75-99 provide justifications for different types of participant classifications such as height, weight, all the way to playing position with no reference on why these are important relative to CLBP.

We have revised the section and added citations throughout.

- Line 126-7: “ it was less effective in capturing nonlinear associations compared to tree-based or kernel-based models.” Any reference for this?

The statement in Lines 126-7 of the Methods on logistic regression's limitations in capturing nonlinear associations compared to tree- or kernel-based models was referred to this study. We rephrased the sentence to remove confusion.

Actions Taken: Rephrased for clarity; added ref (Hosmer et al., 2013) in Methods.

- Overall, please cite proper references as basis of your methodology
We thank Reviewer #3 for the suggestion

Results

- Some of these factors are highly correlated to each other, such as age and level of education within recruited demographics, assuming none of the recruited participants will drop out on elementry. Use of highly correlated variables to create a predictive model can create an unstable model. How come there’s no assessment on the variable correlation?

To address this, we have added a new line about variance inflation factors (all VIF<5), confirming no significant multicollinearity, with a note in Limitations for cautious interpretation of interrelated variables.

Discussion
line 292-296: your results may appear to contradict reference 19, when reference 19 refers to physical education that not all participants received, while your education level may be strongly correlated with age. Receiving education is not equal to receiving physical education.

We have now revised the reference.

Action taken: Compared a Chinese study by Jiang et al., 2025 investigation involving 15,743 people identified 1.24 times greater odds of LBP among those with higher education levels.

6. PLOS authors have the option to publish the peer review history of their article ([what does this mean?](https://track.editorialmanager.com/CL0/https:%2F%2Fjournals.plos.org%2Fdigitalhealth%2Fs%2Feditorial-and-peer-review-process%23loc-peer-review-history/1/010f019950dc805e-d087e8c3-7644-4e1e-9026-1184406b8532-000000/_ZCU4et9Y0VnfDS_dtueHl4P2RgzptVL4YQ9Y3uqhy4=228)). If published, this will include your full peer review and any attached files.

**Do you want your identity to be public for this peer review?** If you choose “no”, your identity will remain anonymous but your review may still be made public.

For information about this choice, including consent withdrawal, please see our [Privacy Policy](https://track.editorialmanager.com/CL0/https:%2F%2Fwww.plos.org%2Fprivacy-policy/1/010f019950dc805e-d087e8c3-7644-4e1e-9026-1184406b8532-000000/BRHxW7o0ptOF0i-40q_QLraNEABmf9GBhc0wdrULujM=228).

Reviewer #1: No

Reviewer #2: **Yes:** Hamidreza Ashayeri

Reviewer #3: No

 [NOTE: If reviewer comments were submitted as an attachment file, they will be attached to this email and accessible via the submission site. Please log into your account, locate the manuscript record, and check for the action link "View Attachments". If this link does not appear, there are no attachment files.]
