# Supplementary material for: Machine Learning-Based Pattern Recognition of Risk Factors for Low Back Pain among Adolescent Cricket Players in Dhaka City
Source: PLOS Digit Health. 2026 Apr 17;5(4):e0001369. doi: 10.1371/journal.pdig.0001369 (PMC13089699; doi:10.1371/journal.pdig.0001369)
Supplement: Attachment — Submitted filename: Rebuttal_Letter_auresp_2.docx [file pdig.0001369.s007.docx]

To,

The Editor, Reviewers,

PLOS Digital Health

Date: 10^th^ February, 2026

**Subject: Addressing Editor/Reviewer Comments for PDIG-D-25-00567R1**

Dear Editor, Reviewers,

Many thanks to you for your helpful feedback. We have now completed a thorough revision of our paper, addressing all of your concerns to the best of our ability. We sincerely apologize for the length of time this process took, but we truly appreciate the helpful feedback you provided. Detailed responses to each of your comments are included below. We welcome any further suggestions you might have to help us improve the work further. The responses are highlighted in blue.

Thank you.

Kind regards,

Authors of PDIG-D-25-00567R1

*Peer Review Analyze: Machine Learning-Based Pattern Recognition of Risk Factors for Low Back Pain among Adolescent Cricket Players in Dhaka City*

PDIG-D-25-00567R1

Machine Learning-Based Pattern Recognition of Risk Factors for Low Back Pain among Adolescent Cricket Players in Dhaka City

PLOS Digital Health

Dear Dr. Marzana Afrooj Ria,

Thank you for submitting your manuscript to PLOS Digital Health. After careful consideration, we feel that it has merit but does not fully meet PLOS Digital Health's publication criteria as it currently stands. Therefore, we invite you to submit a revised version of the manuscript that addresses the points raised during the review process.

Please submit your revised manuscript by Feb 24 2026 11:59PM. If you will need more time than this to complete your revisions, please reply to this message or contact the journal office at digitalhealth@plos.org. When you're ready to submit your revision, log on to https://www.editorialmanager.com/pdig/ and select the 'Submissions Needing Revision' folder to locate your manuscript file.

Please include the following items when submitting your revised manuscript:

* A letter that responds to each point raised by the editor and reviewer(s). You should upload this letter as a separate file labeled 'Response to Reviewers'. This file does not need to include responses to any formatting updates and technical items listed in the 'Journal Requirements' section below.

- Included

* A marked-up copy of your manuscript that highlights changes made to the original version. You should upload this as a separate file labeled 'Revised Manuscript with Track Changes'.

- Included

* An unmarked version of your revised paper without tracked changes. You should upload this as a separate file labeled 'Manuscript'.

- Included

If you would like to make changes to your financial disclosure, competing interests statement, or data availability statement, please make these updates within the submission form at the time of resubmission. Guidelines for resubmitting your figure files are available below the reviewer comments at the end of this letter.

We look forward to receiving your revised manuscript.

Kind regards,

Cleva Villanueva, M.D., Ph.D.

Academic Editor

PLOS Digital Health

Cleva Villanueva

Academic Editor

PLOS Digital Health

Leo Anthony Celi

Editor-in-Chief

PLOS Digital Health

orcid.org/0000-0001-6712-6626

Journal Requirements:

If the reviewer comments include a recommendation to cite specific previously published works, please review and evaluate these publications to determine whether they are relevant and should be cited. There is no requirement to cite these works unless the editor has indicated otherwise.

We have followed this requirement.

Additional Editor Comments:

We appreciate the effort made to revise the manuscript and to address several of the reviewers’ comments. The revised version shows improvements; however, an important methodological concern remains unresolved.

Specifically, the manuscript lacks external validation of the proposed model. While the authors state that they do not currently have the resources to perform external validation and mention this as future work, external validation is a key requirement to ensure the reproducibility, robustness, and generalizability of the results. Even a limited or temporary external validation (e.g., using an independent dataset, a temporal split, or data from a different setting) would substantially strengthen the manuscript.

Without external validation, the conclusions remain insufficiently supported for publication at this stage. We therefore encourage the authors to address this issue directly by providing an appropriate form of external validation or, alternatively, by clearly justifying why such validation cannot be performed and revising the scope and claims of the manuscript accordingly.

For these reasons, the manuscript requires major revision before it can be considered for publication

[Note: HTML markup is below. Please do not edit.]

We sincerely thank the Editor for this crucial direction. We fully agree that external validation is the gold standard for medical AI.

Action Taken: We have performed an external validation using a completely independent dataset of 42 adolescent cricketers from a different region of Bangladesh. Method: We applied our pre-trained SVM model (Sigmoid kernel, C=1, gamma=0.01) to this new dataset without any re-training or hyperparameter tuning. Results: The model achieved a Test Accuracy of 81.0% and a weighted F1-score of 0.81 on the unseen data. Key Finding: Most importantly, the external validation alleviated a concern from our internal cross-validation regarding the "under-detection" of moderate pain. In the external dataset, the Recall (Sensitivity) for Class 2 (Moderate Pain) increased to 79.0% (compared to 66.0% in internal testing), suggesting the model is highly effective at triaging symptomatic players in real-world scenarios.

Revisions:

- We added an "External Validation Cohort" subsection to Materials & Methods.
- We added a new Table 4 in the Results section comparing Internal vs. External performance side-by-side.
- We updated the overall manuscript to reflect these robust findings.

Reviewers' Comments:

Reviewer's Responses to Questions

Comments to the Author

1. If the authors have adequately addressed your comments raised in a previous round of review and you feel that this manuscript is now acceptable for publication, you may indicate that here to bypass the “Comments to the Author” section, enter your conflict of interest statement in the “Confidential to Editor” section, and submit your "Accept" recommendation.

Reviewer #1: (No Response)

Reviewer #2: All comments have been addressed

2. Does this manuscript meet PLOS Digital Health’s publication criteria? Is the manuscript technically sound, and do the data support the conclusions? The manuscript must describe methodologically and ethically rigorous research with conclusions that are appropriately drawn based on the data presented.

Reviewer #1: No

Reviewer #2: Partly

3. Has the statistical analysis been performed appropriately and rigorously?

Reviewer #1: No

Reviewer #2: Yes

4. Have the authors made all data underlying the findings in their manuscript fully available (please refer to the Data Availability Statement at the start of the manuscript PDF file)?

The PLOS Data policy requires authors to make all data underlying the findings described in their manuscript fully available without restriction, with rare exception. The data should be provided as part of the manuscript or its supporting information, or deposited to a public repository. For example, in addition to summary statistics, the data points behind means, medians and variance measures should be available. If there are restrictions on publicly sharing data—e.g. participant privacy or use of data from a third party—those must be specified.

Reviewer #1: Yes

Reviewer #2: Yes

5. Is the manuscript presented in an intelligible fashion and written in standard English?

PLOS Digital Health does not copyedit accepted manuscripts, so the language in submitted articles must be clear, correct, and unambiguous. Any typographical or grammatical errors should be corrected at revision, so please note any specific errors here.

Reviewer #1: Yes

Reviewer #2: Yes

6. Review Comments to the Author

Please use the space provided to explain your answers to the questions above. You may also include additional comments for the author, including concerns about dual publication, research ethics, or publication ethics. (Please upload your review as an attachment if it exceeds 20,000 characters)

Reviewer #1: Thanks for the corrections added but it will be nice if this model was externally validated. This seems like the only missing piece in the puzzle.

We appreciate the reviewer’s positive view of our previous corrections and their sharp identification of the validation gap. We have now provided this "missing piece."

Action Taken: As detailed in the response to the Editor, we successfully validated the model on an external cohort (n=42). The model demonstrated high stability, retaining 100% precision and recall for Class 0 (No Pain), ensuring that no injured players were incorrectly classified as healthy, a critical safety feature for a screening tool. The slight drop in overall accuracy (from 87.6% internal to 81.0% external) is within the expected range for machine learning generalization and confirms the model is not significantly overfitted. These results are now detailed in the Results section (Table 4) and interpreted in the Discussion.

Reviewer #2: I`m grateful for the chance to review the revised version of this manuscript. The authors have addressed the reviewers' comments, and I appreciate their dedication.

They have included essential statistical analyses, such as multicollinearity testing, which enhances the value of their work. They have also applied fundamental revisions in the main text, especially in the discussion section, improving the content of their manuscript.

Their work may prove beneficial for the application of artificial intelligence in low-resource areas.

We thank the reviewer for their encouraging words and for acknowledging the value of our work for low-resource settings. We are glad the inclusion of VIF (Variance Inflation Factor) analysis in the previous round strengthened the statistical rigor.

7. PLOS authors have the option to publish the peer review history of their article (what does this mean?). If published, this will include your full peer review and any attached files.

Do you want your identity to be public for this peer review? If you choose “no”, your identity will remain anonymous but your review may still be made public.

For information about this choice, including consent withdrawal, please see our Privacy Policy.

Reviewer #1: No

Reviewer #2: Yes: Hamidreza Ashayeri

 [NOTE: If reviewer comments were submitted as an attachment file, they will be attached to this email and accessible via the submission site. Please log into your account, locate the manuscript record, and check for the action link "View Attachments". If this link does not appear, there are no attachment files.]
