# Supplementary material for: Machine Learning-Based Pattern Recognition of Risk Factors for Low Back Pain among Adolescent Cricket Players in Dhaka City
Source: PLOS Digit Health. 2026 Apr 17;5(4):e0001369. doi: 10.1371/journal.pdig.0001369 (PMC13089699; doi:10.1371/journal.pdig.0001369)
Supplement: Attachment — Submitted filename: Rebuttal_Letter_auresp_3.docx [file pdig.0001369.s008.docx]

To,

The Editor, Reviewers,

PLOS Digital Health

Date: 14^th^ March, 2026

**Subject: Addressing Editor/Reviewer Comments for PDIG-D-25-00567R2**

Dear Editor, Reviewers,

Many thanks to you for your helpful feedback. We have now completed a thorough revision of our paper, addressing all of your concerns to the best of our ability. We sincerely apologize for the length of time this process took, but we truly appreciate the helpful feedback you provided. Detailed responses to each of your comments are included below. The responses are highlighted in blue.

Thank you.

Kind regards,

Authors of PDIG-D-25-00567R2

*Peer Review Analyze: Machine Learning-Based Pattern Recognition of Risk Factors for Low Back Pain among Adolescent Cricket Players in Dhaka City*

PDIG-D-25-00567R2

Machine Learning-Based Pattern Recognition of Risk Factors for Low Back Pain among Adolescent Cricket Players in Dhaka City

PLOS Digital Health

Dear Dr. Marzana Afrooj Ria,

Thank you for submitting your manuscript to PLOS Digital Health. After careful consideration, we feel that it has merit but does not fully meet PLOS Digital Health's publication criteria as it currently stands. Therefore, we invite you to submit a revised version of the manuscript that addresses the points raised during the review process.

Please submit your revised manuscript by Mar 27 2026 11:59PM. If you will need more time than this to complete your revisions, please reply to this message or contact the journal office at digitalhealth@plos.org. When you're ready to submit your revision, log on to <https://www.editorialmanager.com/pdig/> and select the 'Submissions Needing Revision' folder to locate your manuscript file.

Please include the following items when submitting your revised manuscript:

* A letter that responds to each point raised by the editor and reviewer(s). You should upload this letter as a separate file labeled 'Response to Reviewers'. This file does not need to include responses to any formatting updates and technical items listed in the 'Journal Requirements' section below.

- Included

* A marked-up copy of your manuscript that highlights changes made to the original version. You should upload this as a separate file labeled 'Revised Manuscript with Track Changes'.

- Included

* An unmarked version of your revised paper without tracked changes. You should upload this as a separate file labeled 'Manuscript'.

- Included

If you would like to make changes to your financial disclosure, competing interests statement, or data availability statement, please make these updates within the submission form at the time of resubmission. Guidelines for resubmitting your figure files are available below the reviewer comments at the end of this letter.

We look forward to receiving your revised manuscript.

Kind regards,

Cleva Villanueva, M.D., Ph.D.

Academic Editor

PLOS Digital Health

Cleva Villanueva

Academic Editor

PLOS Digital Health

Leo Anthony Celi

Editor-in-Chief

PLOS Digital Health

orcid.org/0000-0001-6712-6626

Journal Requirements:

If the reviewer comments include a recommendation to cite specific previously published works, please review and evaluate these publications to determine whether they are relevant and should be cited. There is no requirement to cite these works unless the editor has indicated otherwise.

Additional Editor Comments (if provided):

Dear Authors,

Your manuscript has significantly improved, and the revisions have addressed the major methodological concerns raised in the previous round of review. In particular, the addition of an independent external validation cohort (n = 42) substantially strengthens the study and directly resolves the key gap identified earlier. The revised manuscript is now very close to being publication-ready.

Thank you for the thorough and thoughtful revision.

There are, however, a few remaining points that should be addressed prior to acceptance:

1. Reporting uncertainty in external validation

Please include measures of uncertainty for the performance metrics obtained from the external cohort (e.g., 95% confidence intervals via bootstrap methods or exact binomial CIs for class recalls). Given the relatively small sample size (n = 42), performance estimates may be unstable. Reporting confidence intervals will ensure appropriate calibration of the claims. Additionally, consider including the external confusion matrix in the main text or Supplementary Materials.

We thank the Editor for this excellent suggestion to strengthen the reliability of our findings. We have updated the manuscript to include 95% confidence intervals (CIs) for the external validation dataset, which were estimated using bootstrap resampling with 10,000 iterations. These metrics, including the lower and upper bounds, are now detailed in the newly added Table 5. Furthermore, we have included the normalized confusion matrix for the external validation cohort as Figure 3 in the main text to provide clear visual confirmation of the class-wise performance.

2. Clarification of hyperparameter tuning and preprocessing

Please clarify how grid search and preprocessing were conducted relative to cross-validation. If hyperparameters were tuned on the full dataset prior to cross-validation, reported performance may be optimistic. A brief statement confirming that tuning occurred within training folds (e.g., nested cross-validation or equivalent) would resolve this concern.

We appreciate the opportunity to clarify our experimental setup to ensure methodological rigor. We have revised the "Experimental Setup" section to explicitly state that hyperparameter tuning and preprocessing were strictly isolated. Specifically, we noted that hyperparameters were optimized using an exhaustive grid search combined with stratified k-fold cross-validation. We added a clarifying statement confirming that during each fold, hyperparameter combinations were evaluated exclusively on the training subset, while the validation subset was kept completely unseen to prevent any data leakage. Preprocessing steps, such as using StandardScaler, were applied accordingly within this framework.

3. Dataset balancing procedure

The manuscript states that the dataset is balanced (150 samples per class), but the method and stage at which balancing was performed are not fully explained. Please specify whether under-sampling, over-sampling, or class weighting was used. If feasible, consider including a brief sensitivity analysis using the original class distribution (or class weights) to demonstrate robustness.

We thank the Editor for allowing us to clarify this methodology. Data collection was conducted until an equal number of valid instances was obtained for each severity category. Specifically, 150 samples were collected for each class (Class 0, Class 1, and Class 2), resulting in a total cohort of 450 participants. As the dataset was naturally balanced during the data collection phase, no computational over-sampling, under-sampling, or algorithmic class-weighting techniques were required prior to model training. Because this 150-per-class distribution represents our actual, original raw dataset, a sensitivity analysis based on an 'original' imbalanced distribution is not applicable in this context. We have added this clarification to the Materials & Methods section of the revised manuscript.

4. Data Availability statement

There appears to be inconsistent language regarding whether the dataset is fully available within the manuscript/Supplementary Information or “available upon request.” PLOS requires that the underlying data necessary to replicate the results be publicly accessible without restriction, except in rare and justified cases. Please ensure that the final Data Availability statement is fully compliant and consistent throughout the manuscript.

We apologize for the previous inconsistency regarding our data availability. We have corrected the Data Availability statement to fully comply with PLOS guidelines. The manuscript now explicitly states that "All supporting data are available as supporting information". The underlying primary data and the external validation dataset in the S3 Appendix and S4 Appendix files, respectively.

Once these points are addressed, the manuscript should be suitable for publication

[Note: HTML markup is below. Please do not edit.]

Reviewers' Comments:

Reviewer's Responses to Questions

Comments to the Author

1. If the authors have adequately addressed your comments raised in a previous round of review and you feel that this manuscript is now acceptable for publication, you may indicate that here to bypass the “Comments to the Author” section, enter your conflict of interest statement in the “Confidential to Editor” section, and submit your "Accept" recommendation.

Reviewer #1: All comments have been addressed

Reviewer #4: (No Response)

2. Does this manuscript meet PLOS Digital Health’s publication criteria? Is the manuscript technically sound, and do the data support the conclusions? The manuscript must describe methodologically and ethically rigorous research with conclusions that are appropriately drawn based on the data presented.

Reviewer #1: Yes

Reviewer #4: No

3. Has the statistical analysis been performed appropriately and rigorously?

Reviewer #1: Yes

Reviewer #4: Yes

4. Have the authors made all data underlying the findings in their manuscript fully available (please refer to the Data Availability Statement at the start of the manuscript PDF file)?

The PLOS Data policy requires authors to make all data underlying the findings described in their manuscript fully available without restriction, with rare exception. The data should be provided as part of the manuscript or its supporting information, or deposited to a public repository. For example, in addition to summary statistics, the data points behind means, medians and variance measures should be available. If there are restrictions on publicly sharing data—e.g. participant privacy or use of data from a third party—those must be specified.

Reviewer #1: Yes

Reviewer #4: No

5. Is the manuscript presented in an intelligible fashion and written in standard English?

PLOS Digital Health does not copyedit accepted manuscripts, so the language in submitted articles must be clear, correct, and unambiguous. Any typographical or grammatical errors should be corrected at revision, so please note any specific errors here.

Reviewer #1: Yes

Reviewer #4: Yes

6. Review Comments to the Author

Please use the space provided to explain your answers to the questions above. You may also include additional comments for the author, including concerns about dual publication, research ethics, or publication ethics. (Please upload your review as an attachment if it exceeds 20,000 characters)

Reviewer #1: All concerns have been addressed.

We thank the reviewer for their encouraging words.

Reviewer #4: Thank you for the thorough revision. The addition of an independent external validation cohort (n = 42) substantially strengthens the work and directly addresses the key methodological gap raised previously. The revised manuscript is much closer to being publication-ready.

We appreciate the reviewer’s positive view of our previous corrections and their sharp identification of the validation gap.

Key remaining points to address:

1. Please add uncertainty around performance on the external cohort (for example 95% CIs via bootstrap or exact binomial CIs for class recalls). With n = 42, estimates can be unstable, and reporting CIs will make the claims appropriately calibrated. Consider also showing the external confusion matrix in the main text or supplement.

2. Clarify exactly how grid search and preprocessing were conducted relative to cross-validation. If hyperparameters were tuned on the full dataset before CV, reported CV performance may be optimistic. A brief statement confirming tuning within training folds (nested CV or equivalent) would resolve this.

3. The text states the dataset is balanced (150 per class) but does not fully explain how balancing was achieved and at what step. Please specify whether you used under-sampling, over-sampling, or weighting, and consider a short sensitivity analysis using the original class distribution (or class weights) to show robustness.

4. The manuscript contains potentially conflicting language about data being in the manuscript/SI vs “available on request.” PLOS requires the underlying data needed to replicate results to be publicly available without restriction except rare justified cases, so please ensure the final Data Availability statement is fully compliant and consistent throughout.

7. PLOS authors have the option to publish the peer review history of their article (what does this mean?). If published, this will include your full peer review and any attached files.

Do you want your identity to be public for this peer review? If you choose “no”, your identity will remain anonymous but your review may still be made public.

For information about this choice, including consent withdrawal, please see our Privacy Policy.

Reviewer #1: No

Reviewer #4: No

[NOTE: If reviewer comments were submitted as an attachment file, they will be attached to this email and accessible via the submission site. Please log into your account, locate the manuscript record, and check for the action link "View Attachments". If this link does not appear, there are no attachment files.]
